# Region-Specific Associations between Environmental Factors and *Escherichia coli* in Freshwater Beaches in Toronto and Niagara Region, Canada

**DOI:** 10.3390/ijerph182312841

**Published:** 2021-12-06

**Authors:** Johanna Sanchez, Jordan Tustin, Cole Heasley, Mahesh Patel, Jeremy Kelly, Anthony Habjan, Ryan Waterhouse, Ian Young

**Affiliations:** 1 School of Occupational and Public Health, Ryerson University, Toronto, ON M5B 1Z5, Canada; jtustin@ryerson.ca (J.T.); cole.heasley@ryerson.ca (C.H.); iyoung@ryerson.ca (I.Y.); 2 Toronto Public Health, Toronto, ON M5B 2L6, Canada; mahesh.patel@toronto.ca; 3 Niagara Region Public Health, Thorold, ON L2H 0G5, Canada; jeremy.kelly@niagararegion.ca (J.K.); anthony.habjan@niagararegion.ca (A.H.); ryan.waterhouse@niagararegion.ca (R.W.)

**Keywords:** *Escherichia coli*, water quality, recreational water, environmental factors, fecal indicator bacteria

## Abstract

Poor freshwater beach quality, measured by *Escherichia coli* (*E. coli*) levels, poses a risk of recreational water illness. This study linked environmental data to *E. coli* geometric means collected at 18 beaches in Toronto (2008–2019) and the Niagara Region (2011–2019) to examine the environmental predictors of *E. coli*. We developed region-specific models using mixed effects models to examine *E. coli* as a continuous variable and recommended thresholds of *E. coli* concentration (100 CFU/100 mL and 200 CFU/100 mL). Substantial clustering of *E. coli* values at the beach level was observed in Toronto, while minimal clustering was seen in Niagara, suggesting an important beach-specific effect in Toronto beaches. Air temperature and turbidity (measured directly or visually observed) were positively associated with *E. coli* in all models in both regions. In Toronto, waterfowl counts, rainfall, stream discharge and water temperature were positively associated with *E. coli* levels, while solar irradiance and water level were negatively associated. In Niagara, wave height and water level had a positive association with *E. coli*, while rainfall was negatively associated. The differences in regional models suggest the importance of a region-specific approach to addressing beach water quality. The results can guide beach monitoring and management practices, including predictive modelling.

## 1. Introduction

The concentration of *Escherichia coli (E. coli)* is used as an indicator of recent fecal pollution and signifies risks of recreational water illness in freshwater beaches [1]. Municipal beach monitoring programs routinely collect water samples to determine whether bacterial concentrations exceed guideline thresholds. Canadian guidelines implemented in 2012 recommend an *E. coli* geometric mean concentration of less than 200 colony-forming units (CFU)/100 mL averaged from five samples, or less than 400 CFU/100 mL for a single sample, to provide an acceptable risks of illness [2]. Prior to these 2012 guidelines, recreational water quality guidelines recommended a threshold of 100 CFU/100 mL. Despite updated recommendations, Toronto Public Health continues to follow the former and more conservative threshold of 100 CFU/100 mL, while other regions in Ontario have adopted the federal guideline [3]. A limitation of most beach water monitoring programs is the application of a probabilistic approach, where water quality postings are based on the previous day’s testing results due to laboratory processing time (i.e., results typically take 24–48 h to process). Recreational water quality can fluctuate within hours due to a variety of environmental factors, and as such, this approach of water monitoring may not present an accurate picture of current water quality conditions and presents risks of recreational water illness [4,5].

A regional-based approach to understanding the environmental predictors of *E. coli* concentration in fresh waters may help to address gaps in current water monitoring practices and guide the decision-making process. Preceding rainfall, for example, is regularly reported as being a significant predictor of water quality, particularly in urban areas that are influenced by storm water run-off and combined sewage system outfall [5]. Air and water temperature may also play a role in affecting *E. coli* concentrations by influencing the growth and survival of the bacteria [6,7,8]. Wave height and wind speed are also regularly reported as significant in causing changes in concentration, with increased wave height resulting from high wind speed, associated with increased water turbidity [4,9,10]. Higher turbidity results in increased concentration of sediment particles, which facilitates the attachment of *E. coli* and can thus increase their suspension in the water [1,4]. In addition, stream discharge may contribute to increased turbidity and may transport urban runoff to beach areas [1].

In the Toronto and Niagara regions in Ontario, the management of beach water quality is of significant public health importance, with the implementation of an extensive beach monitoring program for the many popular beaches [11]. While there have been studies conducted in several Great Lakes and across North America, few have been conducted in Ontario beaches specifically or in the Canadian context in general [12]. We aim to contribute to this research by examining recreational water quality in these two Southern Ontario regions to identify the environmental predictors associated with *E. coli* concentration at 18 beaches. We aim not to provide a comparative analysis between the regions, but to understand region-specific characteristics while also identifying key trends, to support future beach monitoring activities and the development of predictive models. To achieve this, we link regional public health water quality data with publicly available federal, provincial, and regional environmental data.

## 2. Materials and Methods

### 2.1. Study Area

**Figure 1:**
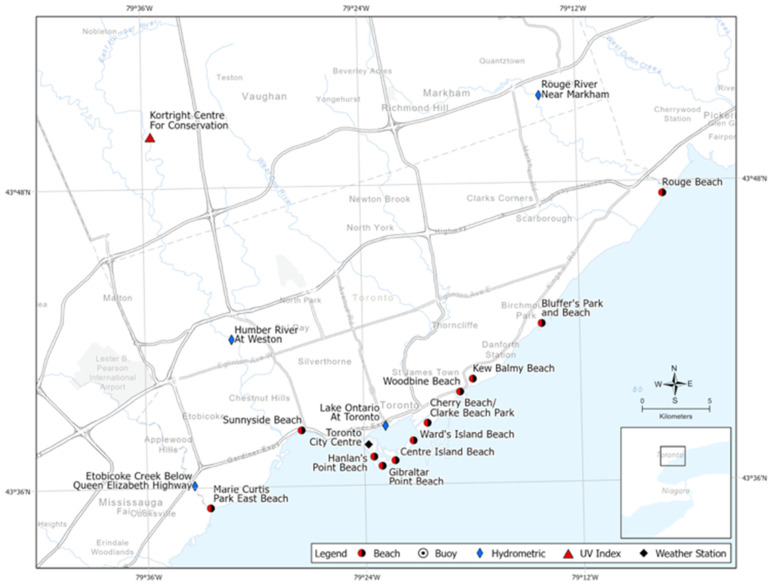

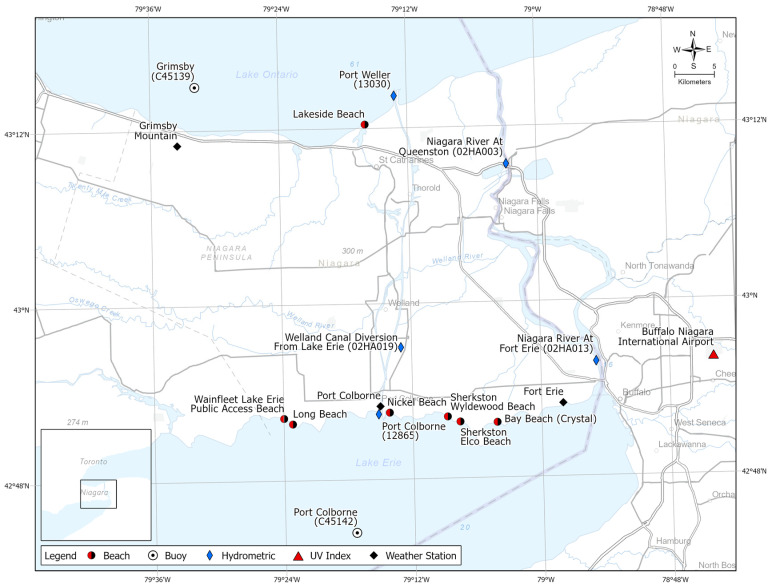
Selected beaches and climate stations in Toronto (2008–2019) and Niagara Region (2011–2019).

We examine the beach water quality in 18 beaches located in two regions in Southern Ontario. Eleven beaches located in Toronto were included in the analysis along with eight beaches in the Niagara Region (Table 1, Figure 1). Of the 28 beaches in Niagara Region, we selected 7 beaches that have been sampled 6 times a week and were popular among beachgoers, while all 11 Toronto beaches were included. Located on Lake Ontario, Toronto is Canada’s most populous city, with a regional population of over 6 million inhabitants, allowing for the opportunity to examine beach water quality in a large urban setting [13]. In addition, Toronto is the leading tourism destination in Canada with over 27.5 million visitors annually [14]. The Niagara Region represents a large geographic area consisting of several municipalities located on both Lake Ontario and Lake Erie, with a total area of 1852 km^2^ and a population of 427,421 [15]. Niagara is also a major tourist destination, receiving 14 million tourists annually [16]. The two regions allow for the exploration of water quality in two of the Great Lakes.

### 2.2. Water Sampling Data

Beach water samples were collected daily at Toronto beaches by the City of Toronto’s Parks, Forestry & Recreation Department (PF&R) from June to the first weekend in September and six times per week at the included Niagara beach sites by Niagara Region Public Health from May to the end of the first weekend in September. Sampling was conducted by Toronto Beach Lifeguards at Toronto beaches, and by public health students in Niagara Region; both the lifeguards and students were trained by public health managers. The Toronto data was collected between 2007 and 2019 and between 2011 and 2019 for the Niagara Region, as provided by the local public health units. Sample collection took place between 7–10 AM each day at knee to waist depth, 15–30 cm below the surface of the water from five pre-specified sampling locations at each beach, following recommended provincial guidelines [3]. Water samples were centrally processed at a Public Health Ontario laboratory within one calendar day of collection using an accredited modified Membrane Filtration method using DC-Agar and an incubation time for Total Coliforms of 24 ± 2 h at 35.5 °C [3,17]. A daily *E. coli* geometric mean for each beach was calculated from the five samples collected.

### 2.3. Environmental Data

Daily precipitation and air temperature (°C) data were obtained from the Canadian Government’s Environment and Natural Resources weather station historical data repository [17]. Toronto data were linked to the Toronto Island weather station for the study period from 2007 to 2019. In Niagara, three weather stations were selected based on completeness of data during the study period (2011–2019): Grimsby Mountain, Port Colborne, and Fort Erie. Beaches in Niagara were linked to one of the three weather stations based on lake location and proximity to the station (Figure 1). Wave height (m) and wind speed (knots) were collected from Environment Canada’s buoy station historical data [18]. Lake Ontario sites were linked to buoy 45,159, while sites located on Lake Erie were linked with buoy 45,142 (Table 2, Figure 1). The Niagara Region water-sampling team also collected shore wave values daily. Stream discharge data were collected from sensors located mid-way through Etobicoke Creek, Humber River and Rouge River in Toronto, and the Niagara River and Welland Canal in the Niagara Region. These data are publicly available on Environment Canada’s streamflow historical data repository [17]. Sites were linked to sensor data based on proximity to the stream. Ultraviolet (UV) radiation data were collected from the closest station collecting these data. In Toronto, solar irradiance data were collected by the Toronto Region Conservation Authority, while in Niagara, the data were linked to UV index data collected by the U.S National Oceanic and Atmospheric Administration’s Weather Service station located in Buffalo, New York, United States [19].

### 2.4. Statistical Analysis

To reduce the skewness of the *E. coli* geometric mean, turbidity, and stream discharge, log transformations were used to satisfy the linear assumptions, prior to data analysis. We then developed region-specific models for Toronto and the Niagara Region using linear mixed effects models and mixed effects logistical models to examine the sources of variation in *E. coli* concentrations. The mixed effects approach was selected for its suitability of addressing a multilevel structure of data, where the data are clustered. In this analysis we expected that *E. coli* concentration values would be clustered at the beach level. We examined the outcome both as a continuous variable of log-transformed *E. coli* concentration and as a binary variable according to two *E. coli* thresholds: 100 CFU/100 mL and 200 CFU/100 mL. These thresholds represent the current federal recreational water quality guidelines, updated threshold recommendation of less than 200 CFU/100 mL, followed by the Niagara region, and the Toronto guideline of less than 100 CFU/100 mL [2]. Separate regional linear and logistical models were developed. Mixed models were fit using Stata version 14.0 [20].

To examine the temporal relationship between environmental conditions and the *E. coli* concentration, we examined values from the previous day for several model covariates, including the previous day *E. coli* concentration, mean weather station air temperature, mean stream discharge, mean wave height, and mean UV index. Rainfall was included as a sum of precipitation (mm) for the two days preceding the day of collection of the water sample. Same-day values of turbidity (Niagara) or water clarity (Toronto), streamflow, wave height, wind speed and waterfowl (Toronto) were included. For the Niagara region, we included both buoy and beach wave height values; however, the final models included only beach shore wave height.

Intercept-only models were developed to explore different levels of variation, without incorporating fixed effects. The beach site was added as the random effect portion of the model to determine if there was a within-group dependence of observations. Each covariate or environmental predictor was added to the model as a fixed effect to assess the significance and suitability for inclusion within the multivariable model. To account for a potential seasonal effect on *E.* *coli* observations, the year was added to the model as a categorical fixed effect. To confirm nesting of the data and the appropriateness of the multilevel method for this analysis, we examined results of intraclass correlation (ICC) tests and the model chi-square. A likelihood ratio test was also used for the Niagara data as an additional confirmatory test.

## 3. Results

### 3.1. Descriptive Data

**Figure 2:**
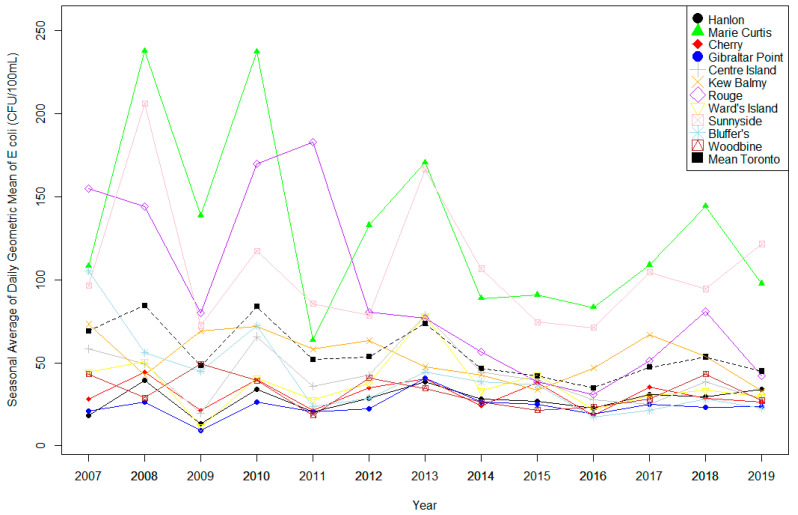
Mean annual *E. coli* geometric mean at Toronto Beaches, 2007–2019.

**Figure 3:**
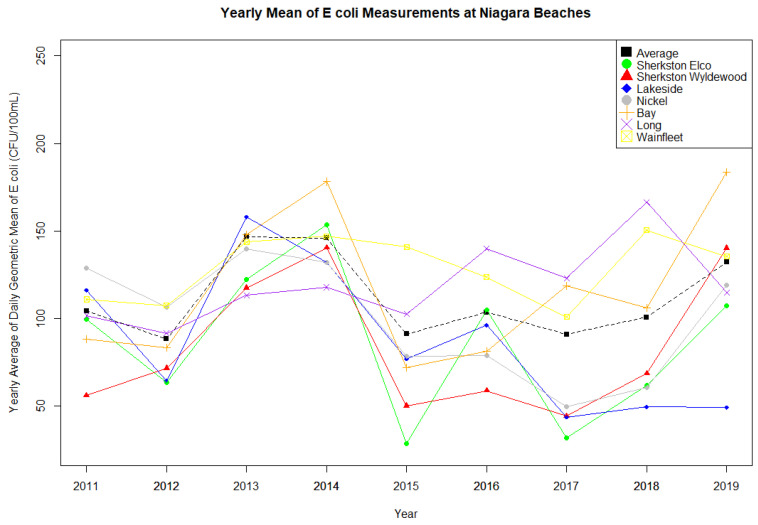
Mean annual *E. coli* geometric mean at Niagara Region Beaches, 2011–2019.

The analysis included 14,324 *E. coli* observations collected between 2007 and 2019 from Toronto and 5149 collected between 2011 and 2019 from Niagara Region. Regional Figure 2 and Figure 3 show the considerable variation in *E. coli* concentrations between the beaches and across the years. In Toronto, the mean annual geometric mean improved during the study period overall, particularly at Marie Curtis, Rouge Beach, and Sunnyside beach (Figure 2, Table S1). In the Niagara Region, geometric means remained fairly consistent across time (Figure 3, Table S2).

**Figure 4:**
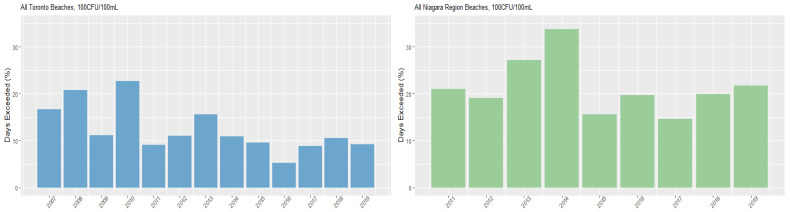
Annual *E. coli* threshold exceedances—100 CFU/100 mL.

**Figure 5:**
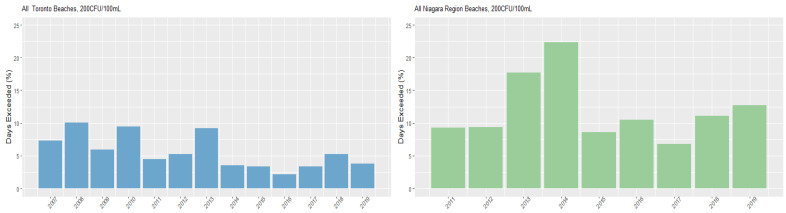
Annual *E. coli* threshold exceedances—200 CFU/100 mL.

Figure 4 and Figure 5 present the percentage of days per season that each region exceeded the 100 CFU/100 mL and 200 CFU/mL health risk thresholds, while Figures S1 and S2 present exceedances by beach. Overall, the Niagara Region had a greater annual exceedance percentage than Toronto beaches. Additionally, as with the overall geometric mean, the proportions of annual exceedances of thresholds varied markedly between the beaches. Marie Curtis and Sunnyside beaches in Toronto present the highest exceedances overall, while Gibraltar Point maintained a low exceedance at both thresholds throughout the study period. Sherkston Elco and Wyldewood beaches had the lowest number of exceedances in the Niagara Region.

Summary statistics for environmental variables are presented in Table S1 for Toronto and Table S2 for Niagara Region. The mean annual summer temperature did not vary significantly between weather stations (Figure S3); however, some variation in seasonal rainfall between weather stations was noted (Figure S4).

### 3.2. Toronto Mixed Effects Models

The random intercepts model of the linear response (continuous measure of *E. coli* concentration), using beach as a random effect, presented a statistically significant chi-square (<0.001), and an intraclass correlation (ICC) of 0.193, suggesting significant clustering of the observations at the beach level, confirming that the multilevel method was the appropriate approach. Similarly, the logistical response intercepts-only models also suggested a multilevel approach for both the 100- and 200-threshold, with significant chi-square (<0.001) for both thresholds and an ICC of 0.206 for the 100-threshold and 0.238 for the 200 CFU/100 mL threshold. Both ICC values suggest significant clustering at the beach and, therefore, a further confirmation of the selected methodological approach.

For all three responses, linear and categorical thresholds, the final model chosen to fit the data contained only variables that had a statistically significant effect on *E. coli* (Table 3). The models showed consistent results and effects of the predictors. The final models using beach as a random effect provided a better fit than the fixed effects model. In the fixed portion of the models, previous day mean temperature, 48 h cumulative rainfall, previous day UV, previous day geometric mean, and stream discharge were positively associated with *E. coli* for all three models. An increased water level was negatively associated with *E. coli* only the linear model. Water clarity was also important, with murky water being positively associated when compared to clear water. A count of 50 or more waterfowl on the beach was positively associated in the linear model and in the 200-threshold model, when compared with no waterfowl on the beach. There was no association between waterfowl and *E. coli* in the 100-threshold model.

### 3.3. Niagara Mixed Effects Models

The random intercepts model of the linear model the ICC of 0.003 did not suggest substantial clustering at the beach level as was seen in the Toronto dataset; however, a likelihood ratio chi-square test was also conducted and demonstrated a model significance at 0.015. Similarly, the intercepts-only logistical model presented an ICC of 0.0131 for the 100-threshold model and 0.004 for the 200-threshold. Again, this indicates low clustering; however, the likelihood-ratio chi-square test was found to be significant in both threshold models, with a chi-square of <0.001 for the 100-thresholds and 0.006 for the 200-threshold.

In the fixed portion of the models, cumulative rainfall in the previous 48 h did not have a statistically significant linear relationship with *E. coli* but had a significant negative effect on both the 100 and 200 thresholds (Table 4). Previous day temperatures, geometric mean and wave height had a positive association with *E. coli* in all three models. Previous day UV index had a negative linear association with *E. coli* in the linear model but was not significantly associated in the 100 and 200-threshold models. Similarly, turbidity had a positive linear association with *E. coli* in the linear model but was not significantly associated in the two threshold models. Finally, increased water level was positively associated with *E. coli* in all models.

## 4. Discussion

This study aimed to explore recreational water quality in two regions in Southern Ontario and evaluate the environmental predictors of fecal contamination as indicated by *E. coli* concentration. We explored this in different formats, both as a linear response, and as a dichotomous outcome, exploring two *E. coli* thresholds—100 and 200 CFU/100 mL. We presented the annual exceedances for each study beach across the two regions and found that while two Toronto beaches had the highest exceedances in the two regions, most Toronto beaches had improved throughout the study period. In 2021, eight Toronto beaches were awarded the Blue Flag designation, a recognized international measure of consistent beach water quality. In the Niagara Region, while there was no worsening of water quality, as indicated by exceedances, there was no evident improvement, and, instead, a plateau was identified in recent years. Lake Erie nutrient concentrations continue to be high and problematic, often resulting in harmful algal blooms, which can lead to hypoxic zones in the water, which has been associated with increased levels of *E. coli* [21,22]. Additionally, increased water levels, wetland deterioration and significant agricultural activities in the region could also be significant contributors to higher *E. coli* levels in the region [21,22].

We identified a difference in the magnitude of the beach effect between the two regions. Initial intercepts-only models identified the substantial clustering of *E. coli* concentrations at the beach level in the Toronto region; however, in the Niagara Region this clustering was minimal. In the final Toronto models, clustering had been addressed by the inclusion of environmental predictors in the model. The strong beach-specific effect in Toronto suggests that water quality studies could benefit from considering each beach as a separate entity with unique characteristics. In Niagara, less variability may be a result of the geographic proximity of most of the included beaches and therefore a similarity in characteristics.

We identified some differences between the two regions and the associated environmental factors. Increased total rainfall in the preceding 48 h was positively associated with increased *E. coli* concentration in both regions. Rainfall is consistently reported as a factor that contributes to increasing *E. coli* concentrations in recreational waters through the resulting increase in surface runoff and storm water discharge [23]. In the Toronto beaches, this is an important consideration that could be associated with the direction of stream flow of the various water systems into Lake Ontario. We examined stream discharge from Etobicoke, Creek, Humber River, and Rouge River into Lake Ontario and found that increased discharge had a positive and significant effect on water quality. Given the urban setting, the relationship with increased rainfall and stream discharge may be an important consideration for Toronto beach water quality. A previous study at Bluffer’s Park Beach in Toronto found that increased *E. coli* concentrations in the stream were associated with rainfall events due to urban runoff [24]. Lakes are closely associated with their watersheds and rivers, therefore, the features of the watershed, which includes size, and surrounding land use, are both directly and indirectly influence water quality and hydrodynamic conditions in the lake [25]. The unique geography of the Niagara Region presents interesting stream flow characteristics, with a north-bound directional flow from Lake Erie to Lake Ontario, flowing primarily through the Welland Canal and Niagara River. In this region, streamflow was not associated with increased *E. coli* concentration even though rainfall was positively associated with the two *E. coli* threshold, suggesting the likelihood of other factors more strongly associated with the exceedance of thresholds in the region.

Water clarity is measured by turbidity, which does not directly measure the number of suspended sediment particles in a sample but instead the absorption and scattering effect that the particles have on light. In Canada, the nephelometric method using NTUs is the recommended method for public health authorities to record this parameter during beach-water sampling. It is recommended that recreational waters remain below 50 NTUs [2]. While turbidity was not measured during the study period in Toronto, a visual observation of the clarity of the water was captured and categorized into clear, mixed, and murky in appearance. As previously described, turbidity was measured at all participating Niagara beaches. Both measures of water clarity were found to have a significant linear relationship with *E. coli* level in both regions, with clear water being negatively associated with *E. coli* concentration in the Toronto beaches, and higher turbidity positively associated with *E. coli* concentration in the Niagara Region. This is consistent with other studies and may be associated with the ability of microorganisms to attach to suspended particles in the water, including organisms suspended from the sediment [1]. In addition, suspended particles may also serve a protective purpose for microorganisms by providing coverage from ultraviolet radiation [2]. Wave activity has also been associated with suspending bacteria from sediments [9]. Increased wave height at the shore had a strong positive linear association with water quality in the Niagara region and was also associated with exceeding the two thresholds. In a study at a Lake Michigan Beach, onshore waves resulted in more active hydrodynamic system, resulting from increased *E. coli* loading from the resuspension of sediment and foreshore sands [26]. Important consideration should be given to open water beaches versus embayed beaches, given the impact of hydrodynamic transport by currents, which is stronger in open water beaches [26].

Ultraviolet radiation was examined using two different measures: solar irradiance, as captured by the Toronto Region Conservation Authority for the Toronto data, and UV index, as captured by a US federal weather station at Buffalo Airport, for Niagara Region. An increase in the value of both types of measures was negatively associated with *E. coli*, both when examined as either a linear response or categorical response. Ultraviolet radiation is a well-described bactericide producing gene damage and inhibiting cell growth [27]. *E. coli* water densities have been found to fluctuate on a 12 h cycle corresponding with the expected periods of maximum and minimum daily level of UV light, with the highest densities reported during the night hours and the lowest densities reported midday [4]. In Toronto, solar irradiance was negatively associated with *E. coli* concentration, whereas in Niagara this was only observed as a linear association, but not as a predictor for exceeding the new thresholds.

Waterfowl fecal contamination is presents a challenge for beach-water quality management as it contains *E. coli* and other enteric pathogens [28]. Birds may deposit feces directly onto the beach, which survive in the sand for a long period and is then released into water by wave erosion and resuspension of bottom sediment [28]. A microbial source tracking (MST) study conducted at Bluffer’s Park Beach found that waterfowl were the main source of contamination of beach water [24]. In our study, waterfowl counts were available for Toronto beaches only, where the presence of more than 50 birds was associated with an *E. coli* concentration greater than 100 CFU/100 mL, whereas the presence of more than 50 birds was associated with 200 CFU/100 mL. Public health authorities should monitor waterfowl and other wildlife in public beaches and should work with other stakeholders to reduce their presence. Targeted MST studies could be useful to determine the relative contribution of waterfowl and other sources of fecal contamination at public beaches to guide potential mitigation strategies.

Finally, previous day temperature and *E. coli* concentration were important positive predictors in all models for the two regions. Air temperature has been associated with influencing water quality by affecting *E. coli* survival and growth, with the bacteria thriving in warmer conditions [7]. Current beach-posting decisions in the Toronto and Niagara Region, as well as many other public health units, are based on previous-day geometric mean results. Our models suggest this is indeed an important factor for current water quality conditions, suggesting that contamination levels may persist, however, there are several environmental variables that also demonstrated other important associations, which could impact water quality conditions. The development of predictive models that incorporate region-specific information in this analysis could be beneficial to beach managers. The models could integrate daily environmental data along with the traditionally used previous day *E. coli* values to predict current conditions and allow public health units to make real-time decisions about beach water quality status.

### Limitations

While several environmental variables were available in the same format between the two regions, different predictor variables were collected by the public health authorities for the two regions. Shore wave height, waterfowl, and turbidity were not collected in both regions. A visual observation of water clarity was available in Toronto as a proxy for turbidity; however, visual observations are subject to bias and data collection inconsistency. In addition, water clarity categories were not provided for data collectors and therefore, a central categorization of individual open-ended classifications was required. Additionally, waterfowl estimation was based only on the presence of birds along the water line during water sampling early in the morning and did not include waterfowl presenting in the dry sandy area or throughout the day. Future research should aim to address these gaps as they could act as important predictors in the respective regions. Selected buoy and weather stations were based on most proximal location and data availability; however, given the distance from many of the study beaches, the data may not accurately represent beach weather conditions. Finally, a further exploration and consideration of hydrodynamic mechanisms responsible for *E. coli* dispersion in freshwater beaches, particularly in different beach structures, such as embayed versus open water, could contribute to the understanding of the interaction between environmental predictors such as rainfall, streamflow, and wind and wave activity.

## 5. Conclusions

Toronto and the Niagara Region represent highly populated areas and popular destinations in Ontario, with beaches spanning two of the Great Lakes. Identifying the environmental factors associated with the recreational water quality in these freshwater beaches could have important public health implications by better informing beach management decisions that impact the health of thousands of beach-goers annually. In this analysis we presented some clear predictor trends between the two regions, as well as some interesting differences. By examining both a linear and categorical response we aimed to understand whether the predictors of exceeding the thresholds were different from the predictors demonstrating a significant linear association. The regional models highlighted some key differences between the two regions, suggesting a region-specific approach is necessary when addressing the factors associated with beach water quality. Additionally, the clustering of Toronto data at the beach level suggests that some important beach-specific characteristics may be important and that beach management practices could be made more effective through the use of targeted beach-specific approaches. The results of this study contribute to the limited research on beach water quality in the Canadian setting and can also be applied to other recreational freshwater settings beyond Southern Ontario. The findings are also important for the development of region- and beach-specific predictive models to support more accurate real-time decisions about recreational water quality safety.

## Figures and Tables

**Table 1:** Selected study beaches in Toronto (2008–2019) and Niagara Region (2011–2019).

Toronto Beaches	Lake	Niagara Beaches	Lake
Bluffer’s Beach Park	Ontario	Bay Beach	Erie
Centre Island Beach	Ontario	Lakeside Beach	Ontario
Cherry Beach	Ontario	Long Beach	Erie
Gibraltar’s Point Beach	Ontario	Nickel beach	Erie
Hanlan’s Point Beach	Ontario	Sherkston Elco Beach	Erie
Kew Balmy Beach	Ontario	Sherkston Wyldewood Beach	Erie
Marie Curtis Park East Beach	Ontario	Wainfleet Lake Erie	Erie
Rouge Beach	Ontario	Public Access Beach	Erie
Sunnyside Beach	Ontario		
Ward’s Island Beach	Ontario		
Woodbine Beach	Ontario		

**Table 2:** Summary of predictors included in analysis from Toronto and Niagara Region.

Variable	Toronto (2007–2019)	Niagara (2011–2019)
Air Temperature	Weather Station	Weather Station, Beach
Rainfall	Weather Station	Weather Station
Solar radiation	Irradiance	UV
Stream Flow	River sensor	River sensor
Turbidity	Lifeguard qualitative observation	Beach
Waterfowl	Lifeguard observation	NA
Water Level	Buoy	Buoy
Water Temperature	Buoy	Buoy, Beach
Wave Height	Buoy	Buoy, Beach
Wind Speed	Buoy	Buoy

**Table 3:** Toronto Linear Mixed Effects and Logistical Mixed Effects Models.

	Linear Response			Categorical Response—100 CFU/100 mL			Categorical Response—200 CFU/100 mL		
	Estimate	SE	*p*-Value	Estimate	SE	*p*-Value	Estimate	SE	*p*-Value
Fixed effects									
48 h total rainfall	0.012	0.001	<0.001	0.027	0.003	<0.001	0.032	0.003	<0.001
24 h air temperature	0.033	0.002	<0.001	0.104	0.013	<0.001	0.090	0.017	<0.001
24 h mean UV	−0.001	0.0001	0.002	−0.003	0.0005	<0.001	−0.003	0.001	<0.001
Log_10_ stream discharge	0.140	0.011	<0.001	0.366	0.050	<0.001	0.248	0.064	<0.001
24 h Log10 *E. coli*	0.242	0.005	<0.001	0.508	0.025	<0.001	0.408	0.026	<0.001
Water level	−0.130	0.059	0.028						
Water clarity									
Clear	ref			ref			ref		
Mixed	0.091	0.057	0.110	0.141	0.273	0.606	0.406	0.369	0.271
Murky	0.367	0.015	<0.001	1.094	0.075	<0.001	1.132	0.107	<0.001
Waterfowl									
0	ref			ref			ref		
1–49	0.032	0.019	0.092	−0.018	0.105	0.865	0.061	0.148	0.679
50–99	0.064	0.027	0.020	0.022	0.144	0.881	0.444	0.194	0.022
≥100	0.104	0.036	0.004	0.088	0.171	0.606	0.497	0.229	0.030
Year									
2008	ref			ref			ref		
2009	−0.080	0.040	0.047	−0.289	0.189	0.128	−0.171	0.243	0.482
2010	−0.089	0.037	0.015	−0.303	0.152	0.047	−0.609	0.202	0.003
2011	−0.279	0.033	<0.001	−1.145	0.174	<0.001	−0.772	0.228	0.001
2012	−0.306	0.040	<0.001	−1.143	0.176	<0.001	−0.912	0.237	<0.001
2013	−0.116	0.034	0.001	−0.633	0.160	<0.001	−0.007	0.205	0.972
2014	−0.170	0.034	<0.001	−0.549	0.163	0.001	−0.692	0.238	0.004
2015	−0.228	0.034	<0.001	−0.697	0.167	<0.001	−0.570	0.243	0.019
2016	−0.253	0.037	<0.001	−1.158	0.215	<0.001	−1.024	0.313	0.001
2017	−0.207	0.053	<0.001	−0.890	0.202	<0.001	−0.514	0.274	0.061
2018	−0.242	0.034	<0.001	−1.195	0.174	<0.001	−0.776	0.230	0.001
2019	−0.142	0.050	0.005	0.597	0.178	0.001	−0.433	0.249	0.082
	Variance	SE		Variance	SE		Variance	SE	
Random effects									
Beach	0.110	0.047		0.848	0.387		0.991	0.445	

**Table 4:** Niagara Models.

	Linear Response			Categorical Response—100 CFU/100 mL			Categorical Response—200 CFU/100 mL		
	Estimate	SE	*p*-Value	Estimate	SE	*p*-Value	Estimate	SE	*p*-Value
Fixed effects									
48 h total rainfall	0.010	0.002	<0.001	0.011	0.006	0.054	−0.009	0.005	0.018
24 h air temp	0.081	0.006	<0.001	0.125	0.013	<0.001	0.099	0.012	<0.001
24 h mean UV	−0.022	0.159	0.040	−0.026	0.388	0.296	0.001	0.022	0.965
24 h Log10 *E. coli*	0.150	0.010	<0.001	0.292	0.024	<0.001	0.287	0.034	<0.001
Water level	0.682	0.015	<0.001	1.919	0.039	<0.001	1.704	0.340	<0.001
Turbidity	0.010	0.001	<0.001	0.007	0.004	0.115	0.003	0.003	0.298
Wave height	0.067	0.003	<0.001	0.076	0.010	<0.001	0.060	0.008	<0.001
Year									
2011	Ref								
2012	0.031	0.093	0.741	0.538	0.228	0.229	0.359	0.197	0.069
2013	0.341	0.082	<0.001	0.559	0.215	0.016	0.551	0.182	0.003
2014	0.361	0.075	<0.001	0.175	0.191	0.129	0.443	0.164	0.007
2015	−0.334	0.079	<0.001	−0.757	0.191	0.004	−0.453	0.166	0.006
2016	−0.128	0.080	0.108	−0.481	0.194	0.024	−0.362	0.167	0.030
2017	−0.276	0.099	0.005	−1.130	0.239	0.004	−0.617	0.207	0.003
2018	−0.304	0.091	0.001	−0.960	0.222	<0.001	−0.676	0.191	<0.001
2019	−0.292	0.127	0.022	−1.220	0.311	<0.001	−0.929	0.271	0.001
	Variance	SE		Variance	SE		Variance	SE	
Random effects									
Beach	0.019	0.012		0.107	0.065		0.051	0.033	

## Data Availability

The environmental data presented in this study are openly available online from the Environment and Climate Change Canada historical data repository (https://climate.weather.gc.ca/historical_data/search_historic_data_e.html) (accessed on 3 August 2020) and the United States NOAA National Weather Service Climate Prediction Centre (https://www.cpc.ncep.noaa.gov/products/stratosphere/uv_index/uv_annual.shtm) (accessed on 13 October 2020). Niagara Region *E. coli* data can be publicly accessed online on Niagara Region Open Data [https://niagaraopendata.ca/] (accessed on 28 July 2020). Toronto *E. coli* data can be requested from Toronto Public Health.

## Supplementary Materials

The following are available online at https://www.mdpi.com/article/10.3390/ijerph182312841/s1, Figure S1: Annual *E. coli* threshold exceedances in Toronto and Niagara Region—100 CFU/100 mL, Figure S2: Annual *E. coli* threshold exceedances in Toronto and Niagara Region—200 CFU/100 mL Table S1: Annual environmental variable values during recreational water sampling season in Toronto, 2007–2019, Table S2: Annual environmental variable values during recreational water sampling season in Niagara Region, 2011–2019.
