# Supplementary material for: Region-Specific Associations between Environmental Factors and Escherichia coli in Freshwater Beaches in Toronto and Niagara Region, Canada
Source: Int J Environ Res Public Health. 2021 Dec 6;18(23):12841. doi: 10.3390/ijerph182312841 (PMC8657392; doi:10.3390/ijerph182312841)
Supplement: Supplementary file 1 [file ijerph-18-12841-s001.zip › ijerph-1412170-supplementary.pdf]

Supplemental Material

Figure S1. Annual *E. coli* threshold exceedances in Toronto and Niagara Region - 100 CFU/100mL

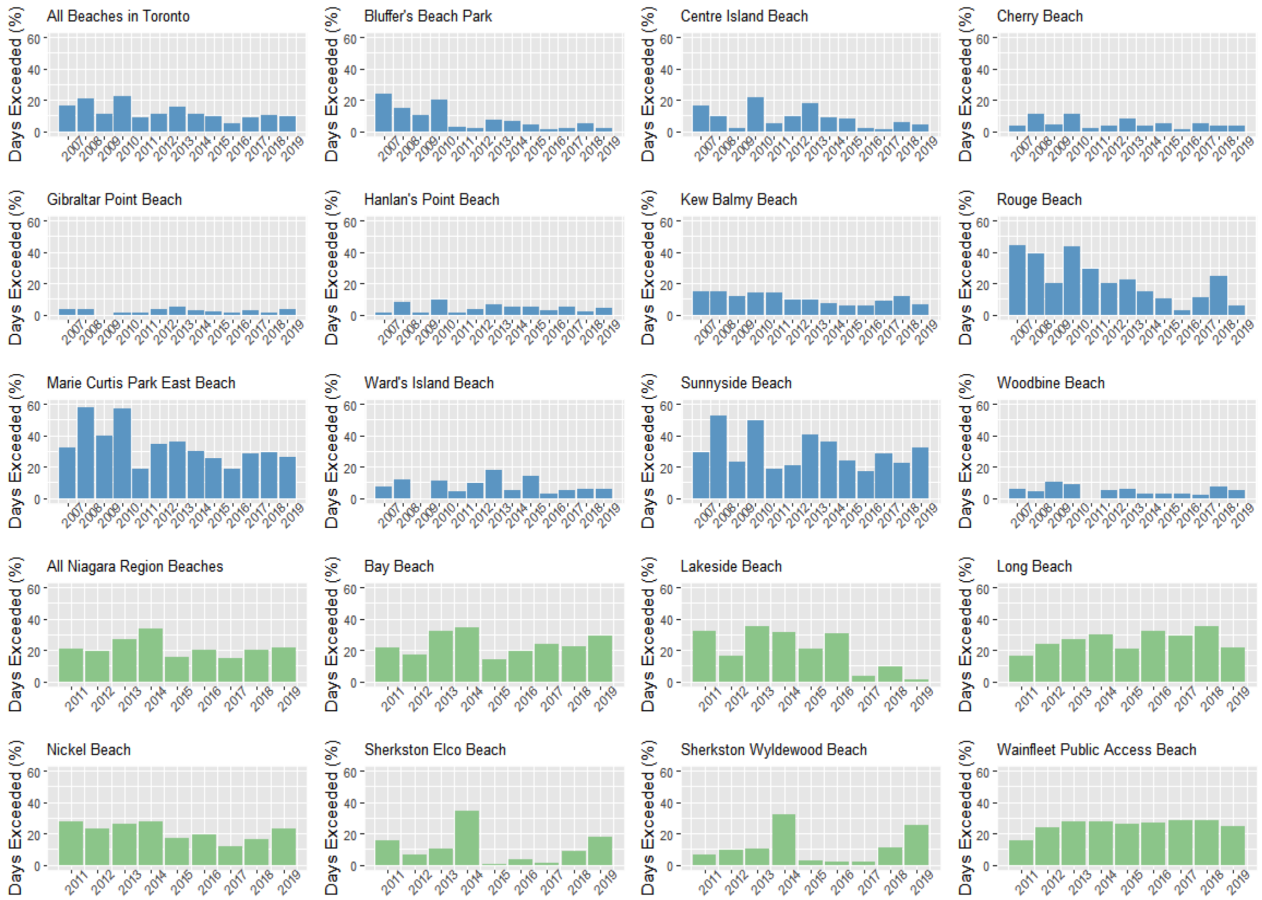

Figure S2. Annual *E. coli* threshold exceedances in Toronto and Niagara Region - 200 CFU/100mL

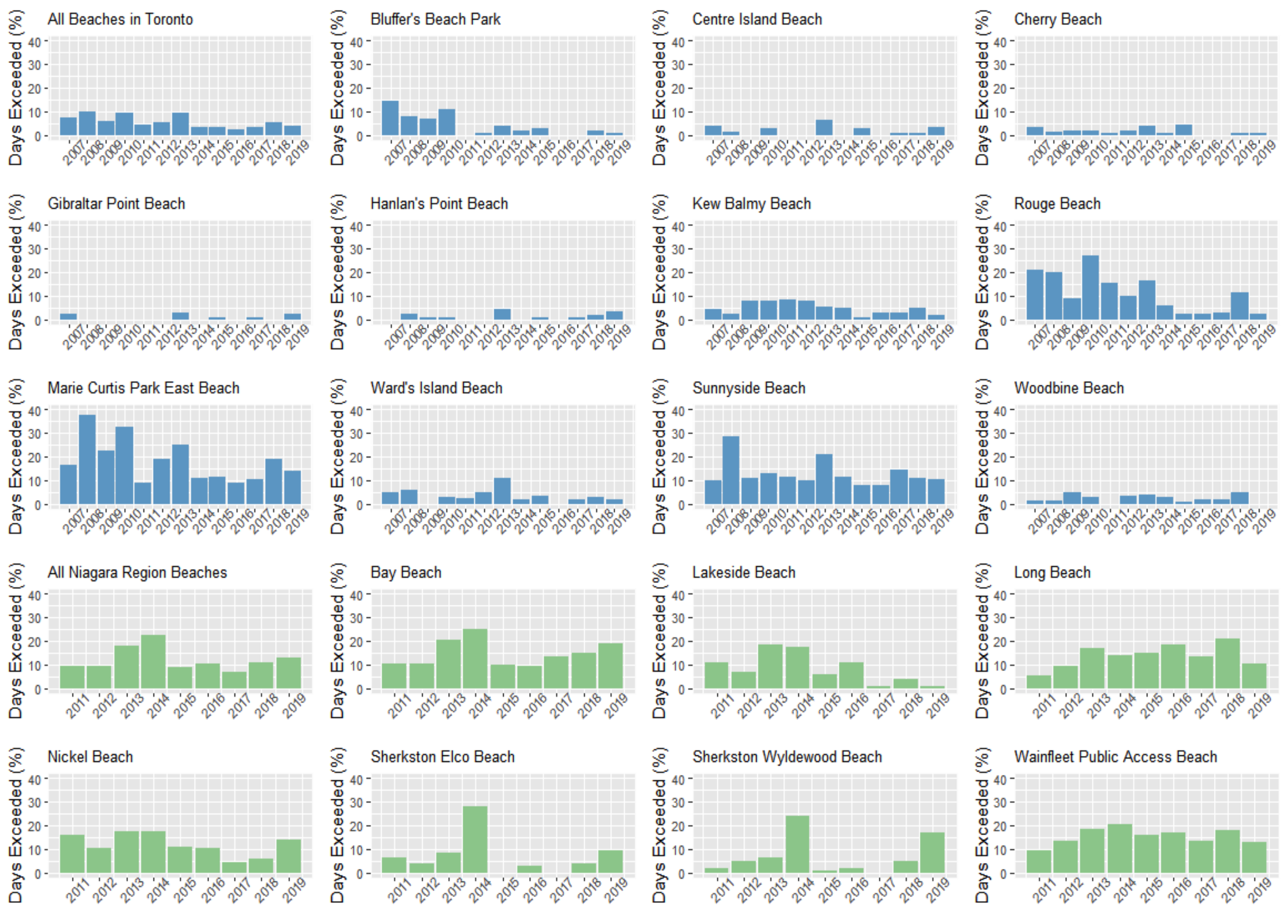

Table S1. Annual environmental variable values during recreational water sampling season in Toronto, 2007-2019

Variable	Total, mean, or median value (±SD)												
	**2007**	**2008**	**2009**	**2010**	**2011**	**2012**	**2013**	**2014**	**2015**	**2016**	**2017**	**2018**	**2019**
Total seasonal rainfall	160	449	415.5	380.3	306.5	374.7	463.1	358.6	264.9	206.9	303.9	385.5	361.4
Mean seasonal air temperature	18.4 (4.4)	17.6 (4.2)	17.3 (4.0)	19.3 (4.5)	19.0 (4.6)	20.0 (4.1)	18.4 (4.3)	17.6 (3.9)	18.1 (3.9)	19.5 (4.9)	17.7 (4)	19.8 (4.5)	18.1 (4.7)
Mean seasonal solar radiation (Kortright station)	NA	960.3 (203.2)	1038 (197.6)	1049.3 (238.3)	1045 (243.6)	1071.7 (209.6)	1047.1 (234.9)	1030.9 (202.3)	1019.3 (228.8)	1097.7 (159.5)	993.1 (288.4)	988.3 (198.9)	996 (239.4)
Mean seasonal water level	74.92 (0.14)	75.19 (0.1)	75.15 (0.07)	74.88 (0.1)	75.18 (0.17)	74.82 (0.12)	75.04 (0.11)	75.12 (0.09)	75.05 (0.16)	74.99 (0.1)	75.7 (0.18)	75.14 (0.15)	75.76 (0.17)
Mean Humber River discharge (m^3/s)	3.44 (5.42)	5.8 (5.7)	7.33 (6.33)	7.59 (9.35)	6.81 (7.73)	4.34 (5.62)	8.99 (17.85)	6.05 (4.9)	6.61 (7.55)	3.02 (2.27)	10.28 (13.77)	4.9 (2.98)	6.73 (6.84)
Mean Rouge River discharge (m^3/s)	0.75 (1.32)	1.88 (1.96)	2.1 (2.24)	2.45 (3.37)	2.16 (2.49)	1.67 (2.22)	2.38 (2.95)	2.15 (2.26)	2.16 (3.62)	0.95 (0.79)	3.41 (5)	1.96 (2.06)	1.81 (1.79)
Mean Etobicoke Creek discharge (m^3/s)	1.47 (3.64)	3.54 (6.44)	3.14 (4.45)	3.39 (5.78)	2.74 (4.2)	1.84 (2.97)	3.71 (7.92)	2.14 (3.01)	2.77 (4.94)	1.24 (2.27)	3.5 (7.25)	2.08 (2.41)	2.57 (3.27)
Mean seasonal count of waterfowl	NA	48 (53.7)	42 (47.9)	48 (66.4)	40 (47)	19 (23.2)	22 (31.2)	28 (41.9)	19 (26.2)	27 (30)	17 (46.9)	11 (21.9)	10 (25.6)

Table S2. Annual environmental variable values during recreational water sampling season in Niagara Region, 2011-2019

	Total, mean, or median value (±SD)								
**Variable**	**2011**	**2012**	**2013**	**2014**	**2015**	**2016**	**2017**	**2018**	**2019**
Total rainfall (mm)	339.7	187.0	476.5	345.8	253.3	161.2	381.2	402.4	372.3
Mean air temperature (˚C)	21.3 (3.5)	22.1 (3.4)	20.3 (3.9)	20.0 (3.4)	19.4 (4.0)	22.0 (3.7)	20.3 (2.9)	22.0 (3.4)	20.2 (3.6)
Mean UV radiation	6.87 (1.91)	6.98 (1.73)	6.40 (1.84)	6.49 (1.53)	6.62 (1.62)	6.88 (1.81)	6.41 (1.79)	6.66 (1.90)	6.87 (1.91)
Median turbidity (NTU)	5.2 (8.4)	4.3 (7.4)	5.0 (6.4)	5.7 (9.0)	4.0 (5.2)	4.4 (6.4)	4.8 (8.4)	3.0 (4.2)	6.5 (12.1)
Mean Niagara River discharge (m^3/s)	6320 (306)	5547 (276)	5752 (256)	6116 (222)	6551 (415)	6567 (297)	7166 (270)	7168 (285)	7850 (272)
Mean Welland Canal discharge (m^3/s)	122 (32.4)	231 (34.7)	175 (48.1)	182 (60.3)	232 (39.0)	233 (39.9)	137 (35.1)	160 (25.5)	NA
Mean Port Colborne Buoy Wave Height (m)	0.4 (0.54)	0.3 (0.40)	0.4 (0.54)	0.3 (0.49)	0.5 (0.58)	0.3 (0.45)	0.2 (0.43)	0.4 (0.50)	0.4 (0.48)
Mean Grimsby Buoy Wave Height (m)	0.3 (0.36)	0.2 (0.27)	0.2 (0.27)	0.3 (0.36)	0.2 (0.36)	0.2 (0.34)	0.1 (0.25)	0.3 (0.36)	0.3 (0.37)
